# A Mesoporous Silica-Based Naringenin Delivery System Promoting Macrophage M2 Polarization in Atherosclerosis

**DOI:** 10.34133/bmr.0248

**Published:** 2025-09-08

**Authors:** Shenhui Ren, Junchao Liu, Hongji Pu, Penghui Wang, Xiaodong Wu, Jinbao Qin, Xiaobing Liu, Minyi Yin, Xinwu Lu, Bo Li, Zhen Zhao

**Affiliations:** ^1^ Department of Vascular Surgery, Shanghai Ninth People’s Hospital, Shanghai Jiao Tong University School of Medicine, Shanghai 200011, China.; ^2^ Department of Vascular Surgery, Institute of Vascular Surgery, Zhongshan Hospital, Fudan University, Shanghai 200032, China.

## Abstract

Atherosclerosis is the leading cause of global cardiovascular morbidity and mortality associated with inflammatory and immunological mechanisms. Immunotherapy has demonstrated promising efficacy in the management of atherosclerosis. Nevertheless, certain immunotherapeutic approaches are associated with limitations, including suboptimal efficacy and non-negligible adverse effects. Upon the pivotal role of macrophage phenotypes in atherosclerosis progression, naringenin-loaded manganese-doped mesoporous silica nanoparticles (MMSN@NAR) were designed and synthesized to reprogram M1 macrophages toward the M2 phenotype, thereby offering a potential therapeutic strategy for atherosclerosis treatment. High loading capacity of naringenin was achieved in MMSN carriers, with superior biocompatibility profiles compared to naringenin dissolved in dimethyl sulfoxide, while maintaining pH-dependent release behavior as demonstrated by dialysis assays. MMSN@NAR is preferentially phagocytosed by M1 macrophages, attenuates inflammatory responses, protects against oxidative stress, and promotes M2 polarization via the AMP-activated protein kinase (AMPK) pathway in vitro. In the ApoE^−/−^ mouse unilateral carotid artery ligation model of atherosclerosis, MMSN@NAR demonstrated marked accumulation in plaques and excellent biocompatibility. Compared to using naringenin or MMSN alone, it could further reduce plaque area by approximately 40% or 60% by inducing macrophage phenotype transformation, which was confirmed by section staining and immunofluorescence. Collectively, this study highlights enhanced macrophage M2 polarization inhibiting atherosclerosis by MMSN@NAR as a promising nanoplatform, offering a novel therapeutic approach based on anti-inflammatory immune regulation.

## Introduction

Atherosclerosis is one of the most prevalent cardiovascular diseases worldwide and serves as the primary etiology of coronary artery disease, stroke, and lower extremity arteriosclerosis obliterans, claiming numerous lives annually [1]. It is considered as a complex inflammatory and immune-mediated disorder rather than a simple age-related degenerative disease [2–4]. Immunomodulatory therapy, by targeting inflammation and immune dysregulation, has become an effective new strategy for treating atherosclerosis [5]. For example, canakinumab achieved notable efficacy by reducing inflammation within atherosclerotic plaques [6]. However, the efficacy of certain immunotherapies has been found to be limited in the treatment of atherosclerosis [7]. Observed challenges include insufficient therapeutic effectiveness, systemic immune suppression due to broad administration routes, and an increased risk of unintended infections. These limitations highlight the urgent need for more targeted delivery approaches with reduced side effects to enhance both the precision and safety of immunotherapy in atherosclerosis management. With the advancement of nanotechnology, nanotherapy has garnered increasing application in the diagnosis and treatment of atherosclerosis [8]. This is attributed to the inherent advantages of nanotherapy, such as its precise targeting capabilities and excellent stability as a drug delivery platform, particularly exemplified by metal–organic frameworks (MOFs) [9,10]. MOFs exhibit superior stability, high drug-loading capacity, cost-effective production, and scalability advantages, making it an ideal nanoplatform for drug delivery.

Abundant macrophages in atherosclerotic plaque play essential roles in the progression of atherosclerosis [11]. The inherent heterogeneity of macrophages within atherosclerotic plaques has been fully elucidated in the complex cellular dynamics of plaque pathogenesis [12,13]. Multiple bioactive mediators, including lipopolysaccharide (LPS), pro-inflammatory cytokines, and low-density lipoprotein (ox-LDL), driving macrophage polarization toward the pro-inflammatory M1 phenotype, exacerbated the local inflammatory milieu and accelerated the progression of atherosclerosis [14,15]. Conversely, M2 macrophages inhibited the progression of atherosclerotic plaques as a protective factor [16,17]. Thus, promoting macrophage M1 polarization toward the M2 macrophage represents a potential novel therapeutic strategy for the treatment of atherosclerosis. This approach has demonstrated efficacy not only in atherosclerosis but also in other conditions such as OA (osteoarthritis) and cancer [18,19]. Nanoparticles such as liposomes and exosomes have been investigated as nanocarriers for macrophage-targeted therapy in atherosclerosis [20,21]. Previous study demonstrated both the feasibility and safety of nanoparticle-mediated drug delivery for modulating macrophage phenotype switching to delay atherosclerosis progression. However, challenges remain regarding nanoparticle drug-loading capacity and circulatory stability in vivo, which require further optimization.

Interestingly, mesoporous silica nanoparticles (MSNs) have emerged as a promising drug delivery systems with exceptional biosafety profile and remarkable versatility in property and structural controllability [22,23]. Furthermore, manganese-doped mesoporous silica nanoparticles (MMSNs) exhibited anti-inflammatory efficacy via effective scavenging of reactive oxygen species (ROS), offering potential therapeutic effects in treating abdominal aortic aneurysms and spinal cord injury [23,24]. Strategic drug loading or surface modification could greatly enhanced the therapeutic outcomes of this nanoplatform [25]. Therefore, the selection of appropriate therapeutic agents for loading has become a critical consideration.

Naringenin, a major citrus flavanone with known anti-inflammatory and antioxidant properties [26], has shown therapeutic potential in abdominal aortic aneurysm and skin inflammation by modulating macrophage phenotypic reprogramming [27–29]. However, its role in atherosclerosis progression remains unexplored. The therapeutic potential of naringenin is hindered by its poor solubility [restricted to dimethyl sulfoxide (DMSO) and methanol] and reliance on oral administration. Hence, a new drug delivery system may address these challenges while improving its targeted accumulation in atherosclerotic plaques.

**Fig. 1.**
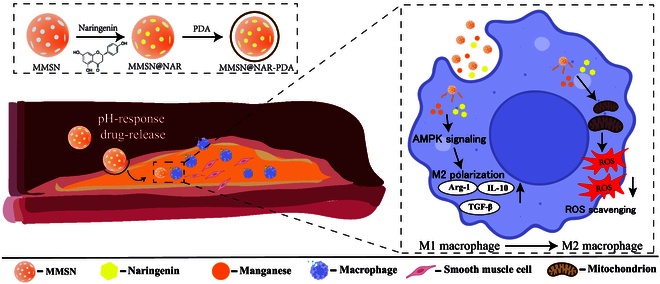
Schematic illustration of the mechanism of the MMSN@NAR nanoplatform for atherosclerosis treatment.

We hypothesized that the combined use of naringenin and MMSN could suppress plaque progression by promoting macrophage polarization toward an anti-inflammatory phenotype. Thus, a novel nanoplatform was designed and synthesized in this study. Leveraging the inherent mesoporous properties of MMSN, the drug naringenin was loaded and the surface was modified with polydopamine (PDA), enabling pH-responsive controlled release of Mn and naringenin. The structure, physical, and chemical properties of the synthesized material were characterized. In vitro, macrophage phenotype switching and enhanced ROS scavenging were observed after material uptake. Moreover, in vivo studies further confirmed its ability to induce macrophage phenotype switching and inhibit the formation of atherosclerotic plaque, demonstrating excellent passive targeting capability and biocompatibility. The biological mechanism of this nanoparticle is presented in Fig. 1.

## Materials and Methods

### Materials

N-(3-dimethylaminopropyl)-N′-ethylcarbodiimide hydrochloride (7084-11-9, 98%, Acmec, China), sulfo-N-hydroxysuccinimide (sulfo-NHS,106627-54-7, 98%, YuanYe, China), lipopolysaccharide (LPS,S1732-0.5mg, Beyotime, China), 5-carboxyfluorescein (5-FAM, 99.5%, Aladdin, China), naringenin (480-41-1, 99.27%, MedChemExpress, China), diethyl pyrocarbonate (1609-47-8, ≥98%, Macklin, China), M-CSF (macrophage colony-stimulating factor, Sigma, USA), CCK-8 kits (Cell Counting Kit-8, BS350A, Biosharp, China), Dulbecco's modified Eagle's medium, and fetal bovine serum (FBS) (Thermo Fisher Scientific, USA) were used.

### Fabrication of MMSN@NAR

MMSNs were synthesized using a modified version of previously reported methods [30,31]. In brief, after multiple steps, the final MMSNs were harvested by centrifugation and subjected to thorough rinsing with both ethanol and water. Based on the experimental protocol, a mixture containing 5 mg of MMSN and 13.61 mg of naringenin was prepared in 5 ml of phosphate-buffered saline (PBS; pH 7.4). The solution was subjected to continuous stirring under dark conditions at an ambient temperature for 12 h. Subsequently, the MMSN@NAR complex was isolated through centrifugation and underwent 3 successive washing cycles with saline solution. For fluorescence labeling, a portion of the obtained material was conjugated with 5-FAM through amid bond formation, which is facilitated by the condensation reaction between amino and carboxyl groups. Finally, 2 mg of PDA was introduced into the MMSN@NAR suspension to complete the fabrication process. Following a 12-h stirring process under light-protected conditions, the resultant products were subjected to centrifugation at 8,000 rpm for 15 min. The obtained material was subsequently washed sequentially with saline solution and deionized water. Finally, the purified products were redispersed in PBS to obtain an MMSN@NAR suspension with a concentration of 1 mg/ml.

### Characterization of MMSN@NAR

The microstructure of MMSN was examined using a JEOL transmission electron microscope (Japan). The release kinetics of Mn and Si ions were monitored through periodic quantification at specified time points (0, 1, 3, 5, 7, 10, 14, 21, 28, and 35 d post-synthesis). Specifically, aqueous suspensions of MMSN (1 mg/ml) were centrifuged at 14,000*g* under ambient conditions at each time point, followed by supernatant analysis using inductively coupled plasma–optical emission spectrometry (ICP-OES; Agilent, USA). Nitrogen adsorption–desorption isotherms and Brunauer–Emmett–Teller (BET) and Barrett–Joyner–Halenda (BJH) methods were applied to determine the pore-size distribution and surface area of MMSN. Zeta potential measurements were performed using a Nicomp Z3000 instrument, with samples dispersed in deionized water (1 mg/ml concentration) at 25.0 °C. The MMSN@NAR nanoparticles were suspended in PBS solution (50 μg/ml) at varying pH levels (6.8, 7.4, and 8.8) and maintained under room temperature conditions with stirring for time intervals of 0, 6, 12, 24, and 48 h. Subsequently, centrifugation was carried out at 5,000*g* for 10 min at 4 °C. The supernatant was collected, and a standard curve was established (0, 2.5, 5, 10, 20, 40, 80, and 160 μg/ml) to quantify the absorbance of these samples at 292 nm using a ultraviolet–visible (UV–Vis) spectrophotometer (Thermo Fisher Scientific, Finland).

### Cell culture

Raw264.7 macrophages and human umbilical vein endothelial cells (HUVECs) were acquired from the Shanghai Cell Bank (Chinese Academy of Sciences). Both cell lines were maintained in high-glucose Dulbecco’s modified Eagle’s medium (DMEM) containing 10% FBS under standard culture conditions (37 °C, 5% CO_2_).

Bone marrow-derived macrophages (BMDMs) were isolated from femurs and tibias of male C57BL/6 mice (6 weeks old) as primary macrophage cultures. Following isoflurane anesthesia, euthanasia was performed via cervical dislocation, after which mice were subjected to 15-min surface sterilization in 70% ethanol. Subsequently, under sterile conditions in a biosafety cabinet, the femurs and tibias were dissected from the mice using autoclaved forceps and scissors.After washing the femurs and tibias three times with PBS (5 min per wash), the bone ends were cut with scissors, and the marrow was flushed out using a 31-gauge insulin needle until no visible red marrow components remained, collecting the cells in high-glucose DMEM supplemented with 10% FBS. The cell suspension was then collected and centrifuged at 1,000*g* for 5 min, after which the cells were seeded at specific densities into 24- and 6-well plates. The cells were cultured in the same medium as used for Raw264.7 cells, supplemented with 10 ng/ml M-CSF. Fresh medium supplemented with 10 ng/ml M-CSF was changed every 48 h over a 7-d period to promote the maturation of primary BMDMs for further experiments.

### Cytotoxicity assay

Raw264.7 macrophages (1 × 10^5^ cells/well) and HUVECs (5 × 10^3^ cells/well) were plated in 96-well plates and cultured for 24 h under standard conditions (37 °C, 5% CO₂, humidified atmosphere). Following initial culture, cells were treated with gradient concentrations of naringenin, MMSN, or MMSN@NAR for an additional 24-h incubation period. Then, the cell viability was measured using a standard CCK-8 assay to evaluate cytotoxicity of the nanoplatform. After incubation in the culture incubator for 30 min, the absorbance at 450 nm was measured using a Multiskan SkyHigh microplate reader (Thermo Fisher Scientific, Finland) to assess cell viability.

### Enrichment of MMSN@NAR in macrophages with different phenotypes

Raw264.7 cells were seeded in a 24-well plate, and M1 polarization was induced by treating them with LPS for 8 h, using PBS as a negative control. Subsequently, MMSN@NAR labeled with 5-FAM fluorescence were added for another 8 h. After washing the cells 3 times with PBS, they were fixed with 4% paraformaldehyde. The cells were permeabilized with 1% BSA (bovine serum albumin) and 1% Triton X-100 for 30 min. The cells were incubated with a primary antibody against β-actin, followed by a Cy3-conjugated goat anti-rabbit secondary antibody. Finally, the nuclei were stained with 4′,6-diamidino-2-phenylindole (DAPI) and the cells were observed under a fluorescence microscope (Eclipse Ni-U, Nikon, Japan).

### RT-qPCR

LPS (2 μg/ml) was used to treat Raw264.7 cells for 8 h and then incubated with different concentration of naringenin, MMSN, or MMSN@NAR. The TRIzol reagent was used to obtain the total RNA. The cDNA was prepared by reverse transcription using the PrimeScript RT Reagent Kit with gDNA Eraser (Perfect Real Time) (Takara, RR047A). The quantitative reverse transcription polymerase chain reaction (RT-qPCR) was conducted on an Applied Biosystems QuantStudio 6 Flex Real-Time PCR System (Thermo Fisher Scientific, USA) using the TB Green Premix Ex Taq (Tli RNaseH Plus) (RR420A, Takara). Three parallel experiments were set up for *Nos2*, *Tnfa*, *Il1b*, *Arg1*, *Il10*, and *Tgfb1*, and glyceraldehyde-3-phosphate dehydrogenase (G*apdh*) was applied as the reference for gene expression calculation via ∆∆Ct method. The primer sequences used in this study were synthesized by BioSune Biotechnology Co. Ltd. (Shanghai, China) and were listed in Table 1.

**Table 1.** Primer nucleotide sequences of qRT-PCR

Name	Primer sequence (5′-3′)
*Gapdh*	F:TCTCCTGCGACTTCAACA
	R:TGTAGCCGTATTCATTGTCA
*Nos2*	F:TACTGCTGGTGGTGACAA
	R:CTGAAGGTGTGGTTGAGTT
*Tnfa*	F:CTGAAGGTGTGGTTGAGTT
	R:ACAAGGTACAACCCATCGGC
*Il1b*	F:CTTCAGGCAGGCAGTATC
	R:CAGCAGGTTATCATCATCATC
*Arg1*	F:AAGGTCTCTACATCACAGAAG
	R:CGAAGCAAGCCAAGGTTA
*Il10*	F:GCTCTTGCACTACCAAAGCC
	R:CTGCTGATCCTCATGCCAGT
*Tgfb1*	F:GGAGAGCCCTGGATACCAACT
	R:TGTGTGTCCAGGCTCCAAAT

### Western blotting assay

LPS-treated Raw264.7 cells were treated with PBS, 100 μM naringenin, 10 parts per million (ppm) MMSN, or 10 ppm MMSN@NAR for 12 h. Protein extraction was performed by cell lysis using a medium radioimmunoprecipitation assay buffer (TargetMol, China) supplemented with a cocktail of protease inhibitors (APExBIO, USA) to prevent protein degradation. The concentration of the proteins was quantified by a BCA (bicinchoninic acid assay) protein assay kit (Thermo Fisher Scientific, USA). Protein samples were denatured at 95 °C for 5 min with 0.2% sodium dodecyl sulfate (SDS) and subsequently resolved by SDS–polyacrylamide gel electrophoresis (PAGE). Subsequently, protein samples were resolved by molecular weight via SDS-PAGE and electrotransferred to polyvinylidene difluoride (PVDF) membranes. After overnight incubation with primary antibodies at 4 °C, membranes were probed with species-matched secondary antibodies. Protein expression was quantified using chemiluminescence detection (GE Healthcare ECL system, USA). Band intensities were semiquantitatively analyzed with ImageJ software [National Institutes of Health (NIH)] and statistically processed using GraphPad Prism, following standardized protocols. Complete antibody specifications for Western blot and immunofluorescence are provided in Table 2.

**Table 2.** Antibodies used in Western blot (WB) or immunofluorescence (IF)

Antibodies	Catalog no. and company	Application	Dilution ratio
iNOS	HY-P80725, MCE	WB	1:1,000
CD86	13395-1-AP, Proteintech	WB	1:1,000
	13395-1-AP, Proteintech	IF	1:100
Arg-1	DF6657-50, Affinity Biosciences	WB	1:1,000
β-Tubulin	10094-1-AP, Proteintech	WB	1:10,000
CD206	SC-70585, Santa Cruz Biotechnology	IF	1:100
β-Actin	BOAR-D0001-B, Biosharp	IF	1:20
p-AMPK	T55608F, Abmart	WB	1:1,000
AMPK	T55326F, Abmart	WB	1:1,000

### Immunofluorescence staining

Circular glass coverslips were placed into a 24-well plate for cell seeding. Raw264.7 and BMDMs were then stimulated with 2 μg/ml LPS to induce polarization toward the M1 phenotype. Subsequently, the cells were treated with PBS, naringenin, MMSN, and MMSN@NAR for 16 h. After treatment, the cells were fixed with 4% paraformaldehyde for 30 min, followed by 3 washes with PBS. Permeabilization and blocking were performed using 1% Triton X-100 diluted in 1% BSA for 30 min. The cells were then incubated with anti-CD86 (1:100, Proteintech, China) and anti-CD206 (1:100, Santa Cruz Animal Health, USA) antibodies at 4 °C overnight. Following primary antibody incubation, the cells were stained with Alexa Fluor 488-conjugated Goat Anti-Rabbit IgG (H+L) (Beyotime, China) and Alexa Fluor Cy3-conjugated Goat Anti-Mouse IgG (H+L) (Proteintech, China) for 1 h. Nuclei were counterstained with DAPI for 5 min, and the cells were observed under a fluorescence microscope. Fluorescence quantification was performed using ImageJ software.

### ROS assay

Intracellular ROS levels were quantified using a ROS assay kit with 2′,7′-dichlorofluorescein diacetate (DCFH-DA) as the fluorescent probe. The Raw264.7 cells were plated on glass coverslips in 24-well plates and first stimulated with LPS for 8 h and then exposed to naringenin, MMSN, and MMSN@NAR for 24 h. Post-treatment cellular staining was performed using DCFH-DA (10 μM, green emission) and DAPI (5 μg/ml, blue emission) for 20 min at 37 °C, with subsequent fluorescence imaging. All procedures were conducted under dark conditions to prevent photobleaching, and experiments were performed in triplicate to ensure reproducibility.

### RNA sequencing and bioinformatics analysis

Raw264.7 cells were seeded in 6-well plates and cultured to 90% confluency. The cells were subsequently treated with either 10 ppm MMSN@NAR or PBS (control) for 8 h. Total RNA extraction was performed using the TRIzol reagent according to the manufacturer’s protocol. RNA library preparation was carried out using the NEBNext Ultra RNA Library Prep Kit for Illumina following the standard workflow. The prepared libraries were then subjected to whole transcriptome sequencing on the Illumina NovaSeq 6000 platform.

For bioinformatics analysis, the raw sequencing reads were initially aligned to the Mus musculus reference genome (GRCm38) using STAR aligner (version 2.7.8). Gene-level read counts were generated using HTseq-count (version 0.13.5) with default parameters. Differential gene expression analysis was performed using DESeq2 (version 1.30.0) in R, with a significance threshold of adjusted *P* value < 0.05. Principal components analysis (PCA) was conducted using the factoextra package in R to visualize sample clustering patterns. Heatmap visualization of differentially expressed genes (DEGs) was generated using the ComplexHeatmap package with hierarchical clustering based on Euclidean distance. Functional annotation of DEGs was conducted to elucidate enriched biological pathways and molecular processes.

### Ligation-induced carotid atherosclerosis in ApoE^−/−^ mouse

The Animal Ethics Committee of Shanghai Ninth People’s Hospital affiliated to Shanghai JiaoTong University School of Medicine approved all animal procedures (approval no. SH9H-2024-A341-SB). Male ApoE^−/−^ mice (6 weeks old) were acclimated for 1 week while maintained on a Western diet prior to surgical procedures. The carotid artery atherosclerosis model was established following established protocols [32].

The experiment included one sham group and 4 experimental groups. In the sham group, after exposing the vessels, the ligature was placed beneath the arteries but was not actually tightened. Following the surgical procedure, the mice were maintained on a Western diet for 5 weeks. Drug administration began at the beginning of the third week after surgery, and the mice that underwent surgery were randomly divided into 4 groups: PBS (control), naringenin (50 mg/kg), MMSN (25 μg/ml), and MMSN@NAR (25 μg/ml). Since naringenin is insoluble in water but soluble in DMSO or methanol, both of which are toxic to the vascular system, we administered the drug via oral gavage. The PBS, MMSN, and MMSN@NAR groups were given via retro-orbital injection (100 μl) per 3 d [33]. Each mouse was weighed weekly. After 3 weeks of drug administration, all mice were euthanized, and blood samples were collected via the enucleation method into 1.5-ml centrifuge tubes. Blood samples were collected in EDTA-coated tubes and maintained at 4 °C until analysis of platelet, red blood cell, and white blood cell counts. After standing at room temperature for 30 min, the blood was centrifuged at 3,500 rpm for 15 min to collect the supernatant and stored at −80 °C, which would be used to assess serum lipid levels and liver/kidney function. Additionally, the aorta, right common carotid artery, heart, liver, spleen, lung, and kidney were harvested and fixed in 4% paraformaldehyde for further analysis.

### Histological staining

After blood sampling, mice were humanely euthanized and perfused transcardially with ice-cold PBS and then fixed by perfusion with 4% paraformaldehyde through the left ventricle. The left common carotid arteries (LCCAs) were then carefully dissected and further fixed in 4% paraformaldehyde. For histological analysis, the samples were processed for paraffin embedding and sectioned for hematoxylin and eosin (H&E) staining and Masson’s trichrome staining. Immunofluorescence analysis was performed on paraffin-embedded carotid artery sections through sequential processing including deparaffinization, antigen retrieval, and blocking with optimized buffers. The sections were then incubated with primary antibodies against CD86 and CD206, followed by appropriate secondary antibodies for fluorescence detection. For each carotid artery specimen, a minimum of 3 representative sections were systematically examined using fluorescence microscopy (Nikon, Japan) at standardized magnifications. Quantitative image analysis was performed using ImageJ software (NIH, Bethesda, MD, USA), with consistent threshold parameters applied for all measurements to ensure comparability across samples. The analysis included quantification of fluorescence intensity and positive staining area, with results expressed as mean ± standard deviation (SD) from 3 independent experiments.

The mouse aortas were fixed in 4% formaldehyde for histological preservation and subsequently subjected to Oil Red O staining to assess atherosclerotic plaque burden. After incubation with Oil Red O staining solution at 37 °C for 30 min, the samples were differentiated using absolute ethanol to achieve optimal staining contrast. The stained aortas were then photographed under bright-field microscopy using a standardized imaging protocol. Quantitative analysis of atherosclerotic plaque area was performed using ImageJ software with the plaque burden expressed as the percentage of Oil Red O-positive area relative to the total aortic surface area.

### Statistical analysis

The data obtained from 3 or more independent experiments are presented as the mean ± SD of the mean. For experimental data with small sample sizes (*n* = 6), we first assessed normality distribution. For data that followed a normal distribution, we employed Student’s *t* test, while for non-normally distributed data and all cases with small sample sizes, we used the Mann–Whitney *U* test. One-way analysis of variance (ANOVA) test was used to compare the differences between multiple groups. The data were analyzed using GraphPad Prism 10.0, and statistical significance was defined using the following thresholds: *P < 0.05, **P < 0.0332, ***P < 0.0021, ****P < 0.0001.

## Results and Discussion

### Preparation and characterization of MMSN@NAR

MMSN@NAR were prepared via 3 steps as previously described [34]. Benefiting from the mesoporous structure of MMSN, naringenin can be effectively encapsulated and subsequently released in a sustained manner, facilitating drug enrichment at the plaque. Functional modification of the nanoparticles was achieved through the conjugation of 5-carboxyfluorescein (5-FAM) for fluorescence tracking and the incorporation of PDA to impart pH-responsive degradation properties, which also enhance the passive target ability of this nanoplatform [35].

**Fig. 2.**
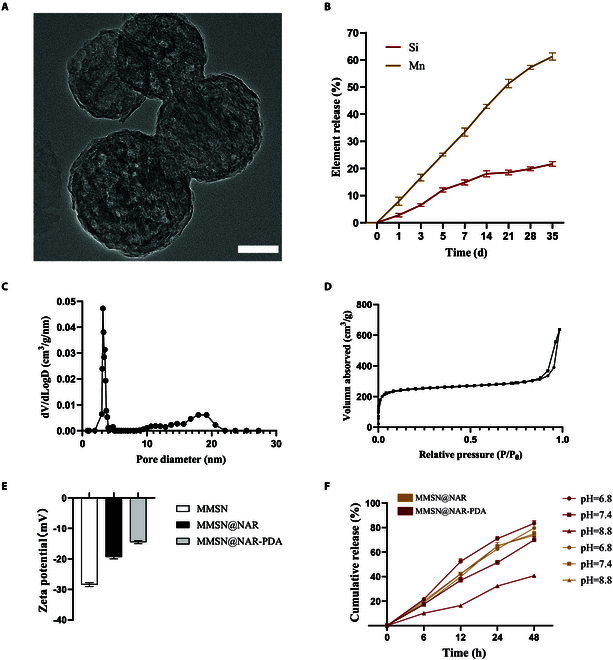
Characterization of MMSN@NAR. (A) TEM image of MMSN. Scale bar, 50 nm. (B) Accumulated element release profiles (*n* = 3) of Mn and Si from MMSN. (C) Mesopore-size distributions of MMSN. (D) N2 adsorption–desorption isotherms of MMSN. (E) Zeta potential of MMSN, MMSN@NAR, and MMSN@NAR-PDA measured at 1 mg/ml in deionized water. (F) Cumulative naringenin release profiles from MMSN@NAR and MMSN@NAR-PDA under varying pH conditions. The concentration of naringenin was determined by measuring the absorbance of samples at 292 nm using a UV–Vis spectrophotometer (*n* = 3).

Primary characterization involved analyzing MMSN morphology and structure using transmission electron microscopy (TEM) coupled with elemental mapping. The nanoparticles displayed uniform spherical morphology with an average diameter of 150 nm (Fig. 2A). The loading efficiency of manganese (Mn) and the release profiles of both Mn and silicon (Si) from mesoporous silica nanoparticles (MMSN) were quantitatively assessed using inductively coupled plasma optical emission spectroscopy (ICP-OES). The Mn ions in the material were released slowly, reaching approximately 32% in the first week and 60% by the sixth week (Fig. 2B). Structural characterization revealed that MMSN possessed a pore size of 9.08 nm with a specific surface area measuring 98.5 m^2^/g (Fig. 2C and D). The material demonstrated favorable mesoporous characteristics, including a well-defined pore size and high surface area, which are instrumental in facilitating efficient drug loading and sustained release kinetics.

Considering the distinctive inflammatory acidic microenvironment within atherosclerotic plaques, we hope to develop a pH-responsive intelligent delivery system to achieve precise drug release and enrichment at the lesion site. PDA, with its acid-responsive degradation capability, serves as an effective functional component for optimizing tissue-specific delivery of nanoplatforms. Upon conjugation with MMSN and PDA, the engineered nanoplatform demonstrated excellent colloidal stability, as evidenced by its positive surface charge with an average zeta potential of −14.50 mV (Fig. 2E). The PDA-modified nanoplatform (MMSN@NAR-PDA) demonstrated enhanced release of naringenin under acidic conditions while showing reduced release in neutral or alkaline environments (Fig. 2F). This pH-responsive release behavior makes it highly suitable for acidic microenvironment in atherosclerotic plaque. These findings highlight the superior drug-loading capacity and pH-dependent release characteristics of MMSN@NAR.

### The optimal concentration and in vitro cellular enrichment of MMSN@NAR

**Fig. 3.**
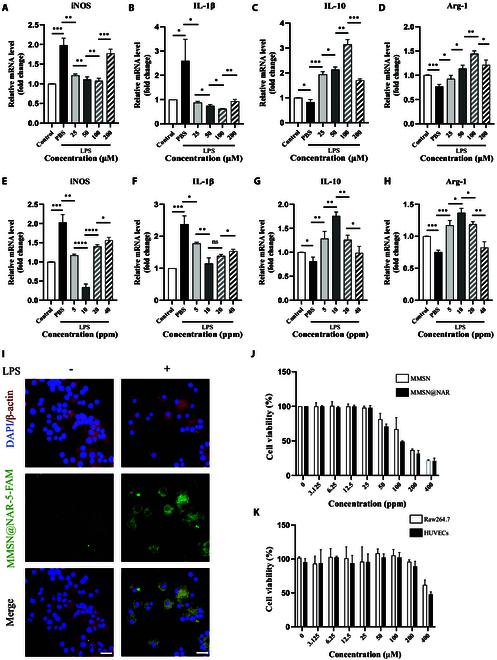
The optimal concentration and in vitro cellular enrichment of MMSN@NAR. qPCR analysis of mRNA expression in Raw264.7 macrophages treated with naringenin at 100 μM (*n* = 3): (A) iNOS, (B) IL-1β, (C) IL-10, and (D) Arg-1. qPCR analysis of mRNA expressions in Raw264.7 macrophages treated with MMSN at 10 ppm (*n* = 3): (E) iNOS, (F) IL-1β, (G) IL-10, and (H) Arg-1. (I) Accumulation of MMSN@NAR on macrophage pretreated by LPS or not. Scale bar, 20 μm. (J) Cell viability of Raw264.7 macrophages incubated with MMSN or MMSN@NAR at different concentrations (*n* = 3). (K) Cell viability of Raw264.7 macrophages and HUVECs incubated with naringenin at different concentrations (*n* = 3). ns, not significant. 0.0332 ≦ *P < 0.05, 0.0021 ≦ **P < 0.0332, 0.0002 ≦ ***P < 0.0021, ****P < 0.0001.

To explore the best concentration of naringenin and MMSN and validate the synergistic role of MMSN@NAR in repolarizing the macrophage phenotype, qPCR analysis was conducted. The results revealed that both MMSN and naringenin exhibited a dose-dependent capacity to induce macrophage phenotypic polarization. Treatment with MMSN (10 ppm) or naringenin (100 μM, equivalent to 27.225 ppm) for 8 h could significantly down-regulate the expression of M1 markers [inducible nitric oxide synthase (iNOS), interleukin-1β (IL-1β)] while up-regulating the expression of M2 markers (Arg-1, IL-10) (all *P* < 0.05, except IL-1β treated with MMSN at 10 ppm and 20 ppm) (Fig. 3A to H). Based on the findings, we synthesized the MMSN@NAR composite with an optimized mass ratio of MMSN to naringenin at a suitable proportion.

As previously mentioned, the M1 macrophage was the mainly type in atherosclerotic plaque, which could further exacerbate the progression of the plaque [14,36]. To investigate the cellular enrichment of MMSN@NAR in M1 macrophages compared to M0 macrophages, we first induced the polarization of macrophages toward the M1 phenotype using LPS. Then, we tested the phagocytic effect of M0 and M1 macrophage phenotypes on MMSN@NAR with 5-FAM labels. Interestingly, we found that MMSN@NAR were more likely to be phagocyted by M1 macrophage (*P* < 0.0021) (Fig. 3I and Fig. S1A). This result may be attributed to the stronger phagocytic function of M1-type macrophages compared to M0-type macrophages, as well as the enhanced permeability and retention (EPR) effect observed in plaques, which is similar to that in cancer [8,37]. Thus, we utilized this characteristic to inhibit the progression of inflammation in atherosclerosis. The above results demonstrate the efficient accumulation of MMSN@NAR in M1-type macrophages in vitro experiment.

To investigate the cytotoxicity of the material in vitro and provide a reference for subsequent animal experiments, the CCK-8 experiment was performed. As demonstrated in the experimental results, MMSN and MMSN@NAR were proved to be safe in Raw264.7 cells and HUVECs (Fig. 3J and Fig. S1B). Furthermore, naringenin revealed minimal cytotoxicity at concentrations below 200 μM in both Raw264.7 and HUVECs, indicating its favorable biocompatibility profile (Fig. 3K). In summary, to ensure optimal therapeutic efficacy while minimizing potential side effects, we selected a concentration of 10 ppm for subsequent cellular experiments, which represents an ideal balance between pharmacological activity and biocompatibility.

### MMSN@NAR-mediated macrophage polarization and ROS elimination in vitro

**Fig. 4.**
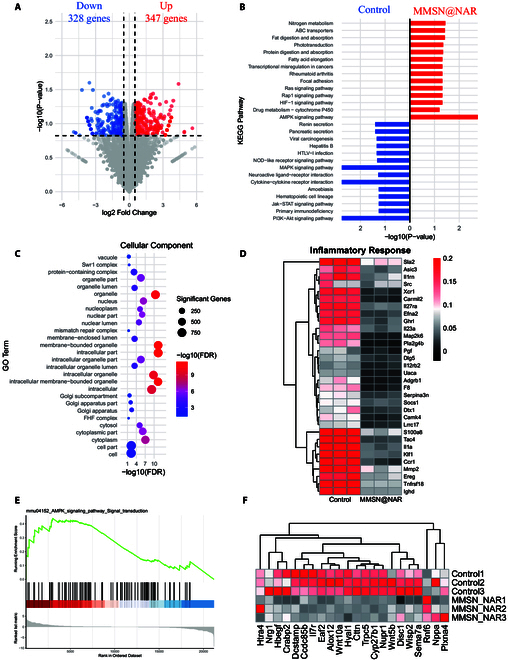
Bioinformatics analysis of IL-1Ra@MMSN effects on macrophages. (A) Volcano plots of the distribution of DEGs detected by RNA-sequencing analysis in Raw264.7 macrophages incubated with MMSN@NAR or PBS (*n* = 3 for each group). The DEGs are determined by cutoff of an adjusted *P* value < 0.05 and a fold change (FC) > 0.5. (B) KEGG pathway clustering of the DEGs. The 14 most significantly up- and down-regulated genes are shown. (C) Clustering of enriched GO-cellular component (CC) for DEG. The GO-CC terms with FDR < 0.05 are shown. (D) Biological insight into the Raw264.7 cells treated with MMSN@NAR. Heatmaps reveal key biological processes in inflammatory response. (E) AMPK signaling pathway (KEGG: mmu04152) is obviously up-regulated in the MMSN@NAR group compared to the control group. (F) Core gene expression heatmap of mTOR signaling pathway.

To further investigate the biological effects of MMSN@NAR on macrophages, we conducted transcriptome sequencing analysis using Raw264.7 cells. Specifically, the cells were first stimulated with LPS for 8 h, followed by treatment with either PBS (control) or MMSN@NAR for further analysis. In comparison with the control group, transcriptomic profiling of the MMSN@NAR-treated group identified 675 DEGs, consisting of 328 down-regulated and 347 up-regulated genes (Fig. 4A). Kyoto Encyclopedia of Genes and Genomes (KEGG) pathway clustering analysis revealed that inflammation-related pathways like nucleotide-binding oligomerization domain (NOD)-like receptor signaling pathway, phosphatidylinositol 3-kinase (PI3K)-AKT pathway, and cytokine−cytokine receptor interaction were substantially down-regulated in the MMSN@NAR group, while AMPK pathway was up-regulated obviously (Fig. 4B). Gene Ontology (GO) pathway clustering analysis found that the genes related to organelle, cellular macromolecule metabolic process, and protein binding were obviously elevated (Fig. 4C and Fig. S2A and B). Based on the results of KEGG and GO analyses, we found that the expression of inflammation-related genes and macrophage pro-inflammatory genes (such as Ereg and Ccr1) in the MMSN@NAR group was reduced, and the response to ROS was weakened (Fig. 4D and Fig. S2C and D). This may be due to the activation of the AMPK signaling pathway and the inhibition of the NOD-like receptor signaling pathway [38]. Reactome pathway clustering analysis revealed the up-regulation of AMPK signaling pathway (Fig. 4E). Following standardized LPS stimulation, Western blot (WB) analysis demonstrated markedly enhanced AMPK phosphorylation in BMDMs treated with MMSN@NAR compared to controls, as revealed by semiquantitative assessment of phospho-AMPK (Thr^172^) levels (Fig. S2E and F) (*P* < 0.0332). Adenosine 5′-monophosphate (AMP)-activated protein kinase (AMPK) is a highly conserved manager, which is sensitive to nutrient and energy balance that is also closely associated to inflammatory response [39]. A previous study has shown that the activation of AMPK could reduce the inflammatory condition in macrophages and thus alleviate the progression of atherosclerosis [40]. We further analyzed the expression of genes within the mTOR (mechanistic target of rapamycin) pathway because the activation of the AMPK pathway is accompanied by the inhibition of the mTOR pathway [41]. As anticipated, many key genes in the mTOR pathway (e.g., Wnt, Hbegf, and Nrg1) exhibited a downward trend in their expression levels (Fig. 4F). In summary, we hypothesize that after treating macrophages with MMSN@NAR, the up-regulation of the AMPK pathway and the down-regulation of the NOD-like receptor signaling pathway and mTOR pathway reduced the inflammatory state in the cells and cleared ROS.

**Fig. 5.**
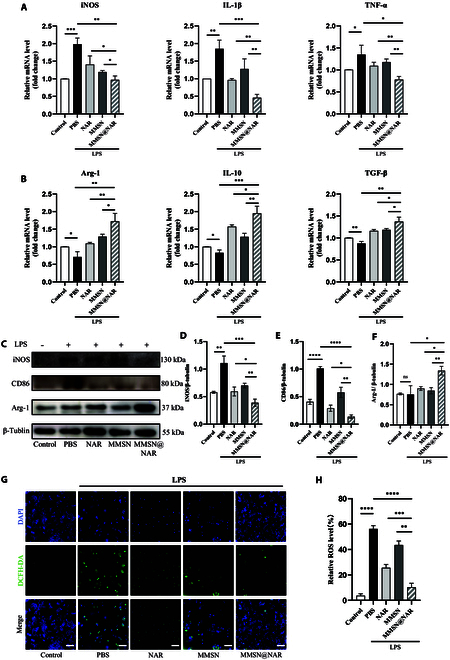
MMSN@NAR-mediated macrophage polarization and ROS elimination in vitro. qPCR analysis of mRNA expression in Raw264.7 macrophages treated with MMSN@NAR at 10 ppm (*n* = 3). M1-related pro-inflammatory gene expression: (A) iNOS, IL-1β, and TNF-α. M2-related anti-inflammatory gene expression: (B) Arg-1, IL-10, and TGF-β. (C) Western blot analysis of iNOS, CD86, and Arg-1 protein levels in BMDM cells. Semiquantitative analysis of (D) iNOS, (E) CD86, and (F) Arg-1 expression levels in the indicated groups (*n* = 3). (G) Representative images of the intracellular ROS levels detected by oxidant-sensing probe DCFH-DA (green) in Raw264.7 macrophages. Scale bar, 100 μm. (H) Quantification of ROS levels in the indicated groups. Data shown are representative of 3 independent experiments. 0.0332 ≦ *P < 0.05, 0.0021 ≦ **P < 0.0332, 0.0002 ≦ ***P < 0.0021, ****P < 0.0001.

To validate the effort of MMSN@NAR on the macrophage phenotype transformation, we first pretreated the Raw264.7 cells with LPS to induce their phenotypes prone to M1, and then MMSN@NAR and controls were added into the culture medium for another 8 h. To exclude potential effects of the solvent DMSO used for naringenin, we prepared a 100 mM naringenin stock solution in DMSO and diluted it 1:1,000 to ensure that the final DMSO concentration remained below 0.1%. In all other experimental groups, we added an equivalent volume of DMSO alone to control for any potential solvent effects on cellular status. Following the extraction of mRNA from cells, qPCR was performed to analyze the expression of pro-inflammatory and anti-inflammatory genes in Raw264.7 cells. The results revealed a significant down-regulation of pro-inflammatory gene expression [iNOS, IL-1β, and tumor necrosis factor-α (TNF-α)], accompanied by a marked up-regulation of anti-inflammatory gene expression [Arg-1, IL-10, and transforming growth factor-β (TGF-β)] (all *P* < 0.05) (Fig. 5A and B). Furthermore, Western blot analysis was conducted to validate the expression of macrophage-related markers at the protein level in both Raw264.7 and bone marrow-derived macrophages (BMDMs) (Fig. 5C to F and Fig. S3A). The results demonstrated that the treatment could effectively suppress the expression of CD86 and iNOS while up-regulating Arg-1, a characteristic marker predominantly associated with M2 phenotype macrophages (all *P* < 0.05 in group MMSN@NAR versus group LPS, group NAR, and group MMSN). The results were also confirmed by immunofluorescence (Fig. S4B to D). The results revealed that after treatment with MMSN@NAR for the same duration, the ratio of CD206 to CD86 expression on the surface of macrophages was significantly increased compared to the groups treated with MMSN or NAR alone, indicating the phenotype transformation of macrophages. These findings highlight a promising synergistic interaction between MMSN and naringenin, underscoring the potential for improved anti-inflammatory and M2 polarization outcomes.

The outcomes of transcriptome sequencing analysis and the phenotypic transformation of macrophages prompted us to investigate whether their functional properties were also altered. To address this, we evaluated the capacity of macrophages to eliminate ROS. We found that the ROS levels in the MMSN@NAR-treated group were significantly reduced compared to the other treatment groups (all *P* < 0.0332) (Fig. 5G and H). This finding suggests that the phenotypic shift in macrophages not only reflects their polarization but also enhances their anti-inflammatory capabilities, further supporting the therapeutic potential in treating atherosclerosis.

### Biocompatibility and plaque enrichment of MMSN@NAR in vivo

**Fig. 6.**
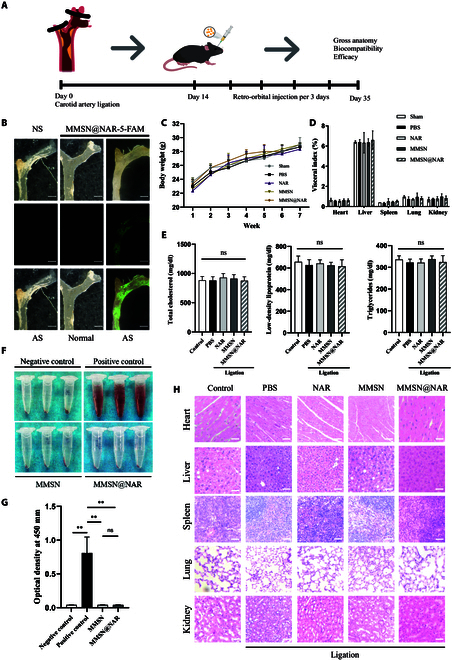
Biocompatibility and plaque enrichment of MMSN@NAR in vivo. (A) Schematic diagram of the experimental procedure in vivo. (B) Accumulation of MMSN@NAR in atherosclerotic plaques in vivo. Scale bar, 1 mm. (C) Body weight of mice during experimental procedure (*n* = 6). (D) Visceral index of mice at day 35 (*n* = 6). (E) Content of total cholesterol, LDL cholesterol, and triglycerides in mouse serum (*n* = 6). 0.0332 ≦ *P < 0.05, 0.0021 ≦ **P < 0.0332, 0.0002 ≦ ***P < 0.0021, ****P < 0.0001.

The efficiency of MMSN@NAR in vitro experiment motivated us to further explore its potential in vivo. We used 6-week-old C57/B6 male ApoE^−/−^ mice fed a Western diet. During the first week after arrival, the mice were fed a mixed diet of regular chow and high-fat diet to allow them to acclimate to the environment. To accelerate the development of atherosclerosis, the carotid artery was ligated with a fine thread without interfering with the blood flow of the inferior thyroid artery. Two weeks after the ligation, MMSN@NAR were injected through retro-orbital injection every 3 d for another 3 weeks (Fig. 6A). Throughout the experimental period, no significant differences were observed in food consumption, body morphology, coat condition, or behavioral patterns between the naringenin, MMSN, and MMSN@NAR groups when compared to either PBS or sham control groups. To investigate whether MMSN@NAR could accumulate in atherosclerotic plaques in vivo, we injected mice with 5-FAM-labeled MMSN@NAR compounds. Our findings revealed pronounced accumulation of the material within the atherosclerotic plaques of the carotid arteries, indicating a high concentration of nanoparticles in the plaques (Fig. 6B). At the end of the experiment, the mice were subjected to excessive anesthesia and blood was collected via the enucleation method. The carotid artery on the ligated side and various organs was subsequently harvested for further experimental analysis. No significant difference in body weight was found between the groups (all *P* > 0.05) (Fig. 6C). Neither MMSN, naringenin, nor MMSN@NAR affect the level of total cholesterol, LDL, or triglycerides in the blood (all *P* > 0.05) (Fig. 6E). Hemocompatibility assessment demonstrated negligible hemolytic activity in the MMSN@NAR group, indicating excellent blood stability and suitability for retro-orbital administration (*P* < 0.05 compared to positive control) (Fig. 6F and G). Additionally, the counts of red blood cells, white blood cells, and platelets, as well as hepatic and renal function tests, showed no significant differences across the experimental groups (all *P* > 0.05) (Fig. S4). These results suggest that the material exhibits excellent biocompatibility, with no adverse effects on blood composition or physiological functions. We also measured the organ weight-to-body weight ratio and performed H&E staining on various organs to evaluate the effects of the material on the development of mouse organs in vivo. No significant differences were observed in the visceral-to-body weight index of the mice (all *P* > 0.05) (Fig. 6D). Histopathological analysis via H&E staining revealed no structural alterations in the heart, liver, spleen, lungs, or kidneys. Additionally, there was no evidence of cellular atrophy, necrosis, or fibrosis in any of the examined tissues (Fig. 6H). In summary, the results collectively demonstrate that the nano-drug delivery system exhibits favorable biocompatibility and minimal toxicity in vivo.

### Inhibited atherosclerosis plaque progression by MMSN@NAR

**Fig. 7.**
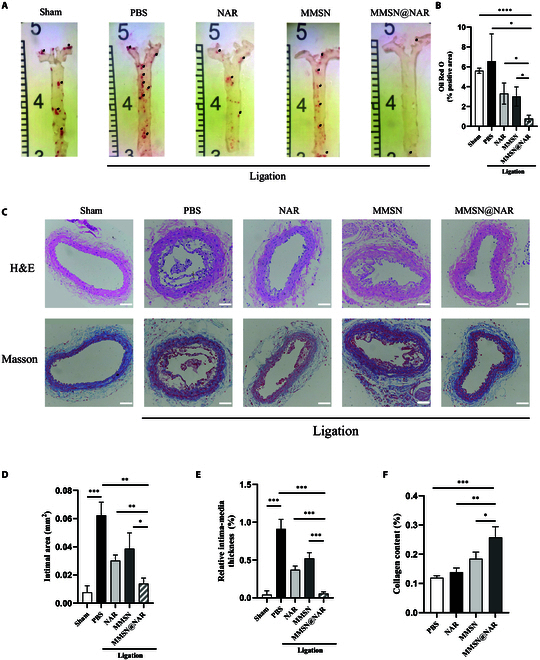
Inhibited atherosclerosis plaque progression by MMSN@NAR. (A) Oil Red O staining of whole aortas. Black arrows indicate Oil Red O-positive areas (*n* = 6). (B) Percentages of Oil Red O-positive areas among whole aorta areas. (C) H&E staining and Masson staining of the atherosclerotic plaques in carotid arteries. Scale bar, 100 μm. (D) Measured absolute area of intimal hyperplasia. (E) Intima-to-media ratio. (F) Collagen content in the plaque assessed by Masson staining. 0.0332 ≦ *P < 0.05, 0.0021 ≦ **P < 0.0332, 0.0002 ≦ ***P < 0.0021, ****P < 0.0001.

Following a 3-week treatment regimen with MMSN@NAR or other experimental reagents, mouse aortic and carotid artery tissues were harvested and subsequently subjected to histological staining using both H&E and Masson staining. Mice aortas were stained with Oil Red O reagent to observe the overall plaque burden in mice. We found that mice treated with MMSN@NAR could have much mild atherosclerosis burden compared to the mice in other groups (all *P* < 0.05) (Fig. 7A and B). Histological analysis with H&E and Masson staining revealed that carotid artery ligation strongly induced plaque formation at the ligation site. Both naringenin and MMSN exhibited inhibitory effects on atherosclerosis, while MMSN@NAR reduced the plaque area by nearly 40% and 60% compared to using naringenin or MMSN separately, demonstrating clearly superior outcomes compared to their individual application (Fig. 7C). Analysis of the absolute area of the arterial intima and the intima-to-media area ratio revealed that the MMSN@NAR group exhibited a more favorable suppression of arterial intimal hyperplasia (all *P* < 0.05) (Fig. 7D and E), as well as relative plaque area in the carotid artery (Fig. S5). The stability of atherosclerotic plaques in human plays a critical role in determining the risk and prognosis of cardiovascular events [42]. M2-polarized macrophages exert atheroprotective effects, which not only attenuate plaque progression but also enhance plaque stability by promoting fibrous cap formation and extracellular matrix remodeling [14]. Therefore, we further analyzed the collagen fiber content in the plaques; as expected, the MMSN@NAR group exhibited significantly higher collagen fiber content, suggesting enhanced plaque stability (all *P* < 0.05) (Fig. 7F). However, since the material itself possesses a robust ability to inhibit plaque formation, this finding should be interpreted with caution and requires further investigation to validate our observations.

**Fig. 8.**
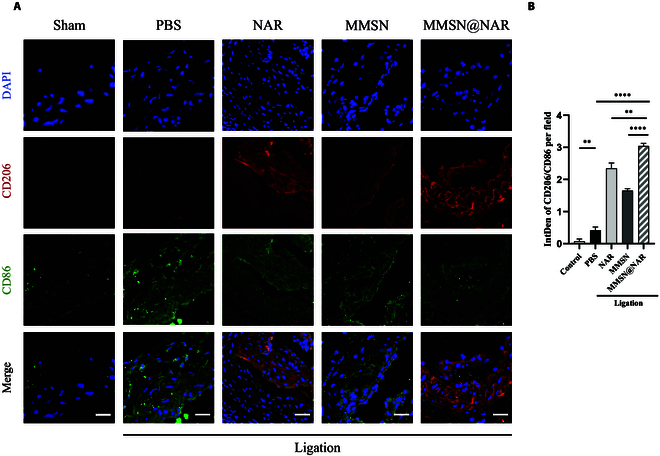
Immunofluorescent staining of carotid arteries from the mouse model. (A) Representative images of CD206 (red) and CD86 (green) costaining of carotid arteries from the sham group (no ligation) and the PBS, naringenin, MMSN, and MMSN@NAR groups (all subjected to ligation surgery) (*n* = 3). Scale bar, 200 μm. (B) CD86/CD206 ratio as a measure of M1/M2 macrophage polarization in the plaque. 0.0021 ≦ **P < 0.0332, ****P < 0.0001.

In the end, to investigate whether the phenotype of macrophages within atherosclerotic plaques had changed, immunofluorescence staining was performed. In the MMSN@NAR group, the ratio of positive signals for M2-type macrophages to M1-type macrophages was significantly higher, indicating an increased proportion of M2-type macrophages within the plaques (Fig. 8A and B). Subsequently, we conducted immunohistochemical staining for proteins secreted by M1 and M2 macrophages to validate our findings. Overall, the level of Arg-1, secreted by M2 macrophages, was elevated in the MMSN@NAR group, whereas the level of iNOS, secreted by M1 macrophages, was reduced (Fig. S6). In summary, the increased proportion of M2 macrophages within atherosclerotic plaques effectively inhibits plaque progression, as facilitated by MMSN@NAR.

Compared to alternative nanoplatforms, our system demonstrates superior characteristics including simplified fabrication procedures, versatile surface modification capacity, high drug-loading efficiency, excellent biocompatibility, and enhanced therapeutic efficacy, indicating strong potential for biomedical applications. However, this study has several limitations. First, we relied solely on a mouse model without further validation in other animal species. Second, the current material predominantly accumulates in plaques via passive targeting, which may lead to suboptimal delivery efficiency. Future studies will focus on surface modifications to achieve active targeting for enhanced precision and therapeutic efficacy.

## Conclusion

In summary, MMSN@NAR was successfully designed and fabricated. The newly developed nanoplatform exhibited superior drug encapsulation capacity, remarkable colloidal stability, and pH-dependent release characteristics. MMSN@NAR exhibited remarkable accumulation in M1 macrophages. The release of naringenin and manganese (Mn) promoted the repolarization of M1 macrophages to the M2 phenotype and protected against oxidative stress injury by activating the AMPK signaling pathway. In vivo, MMSN@NAR exhibited excellent accumulation in atherosclerotic plaque, hemocompatibility, and biocompatibility. Furthermore, this nanoplatform effectively reduced the progression of atherosclerosis by inducing macrophage reprogramming within the plaque, facilitating macrophage phenotype transformation, and potentially improving plaque fibrosis and stability, thereby reducing the risk of cardiovascular events. This study presents an anti-inflammatory strategy for early atherosclerosis intervention, demonstrating MMSN@NAR as an effective nanoplatform for targeted therapy.

## Data Availability

The data that support the findings of this study are available from the corresponding author upon reasonable request.
