# Supplementary material for: A Mesoporous Silica-Based Naringenin Delivery System Promoting Macrophage M2 Polarization in Atherosclerosis
Source: Biomater Res. 2025 Sep 8;29:0248. doi: 10.34133/bmr.0248 (PMC12415335; doi:10.34133/bmr.0248)
Supplement: Supplementary 1 — Figs. S1 to S6 [file bmr.0248.f1.docx]

**Supporting information**

**for**

**Mesoporous Silica-Based Naringenin Delivery System Enhances Macrophage Polarization for Effective Atherosclerosis Management**

Shenhui Ren ^a,#^, Junchao Liu ^a,#^, Hongji Pu ^a^, Penghui Wang ^b^, Xiaodong Wu ^a^, Jinbao Qin ^a^, Xiaobing Liu ^a^, Minyi Yin ^a^, Xinwu Lu ^a,*^, Bo Li ^a,*^, Zhen Zhao ^a,*^

**Supplementary Figures and Figure Legends**

**
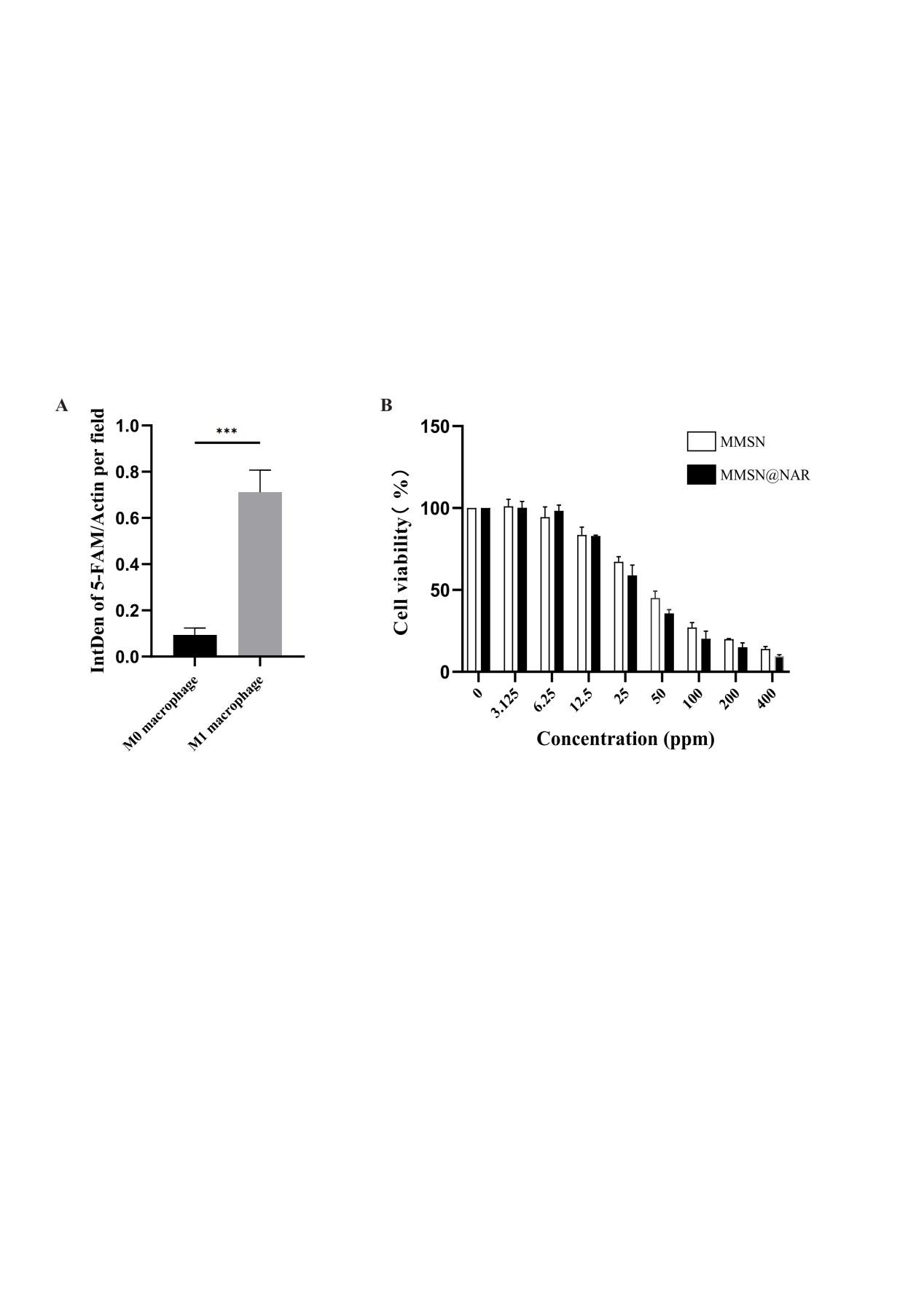
**

Supplementary Fig.1. The optimal concentration and in vitro targeting ability of MMSN@NAR. A)The fluorescence intensity of 5-FAM-labeled MMSN@NAR in M0 and M1 macrophages cells. B) Cell viability of HUVECs incubated with MMSN or MMSN@NAR at different concentrations (n = 3)..0.0002≦***p＜0.0021.


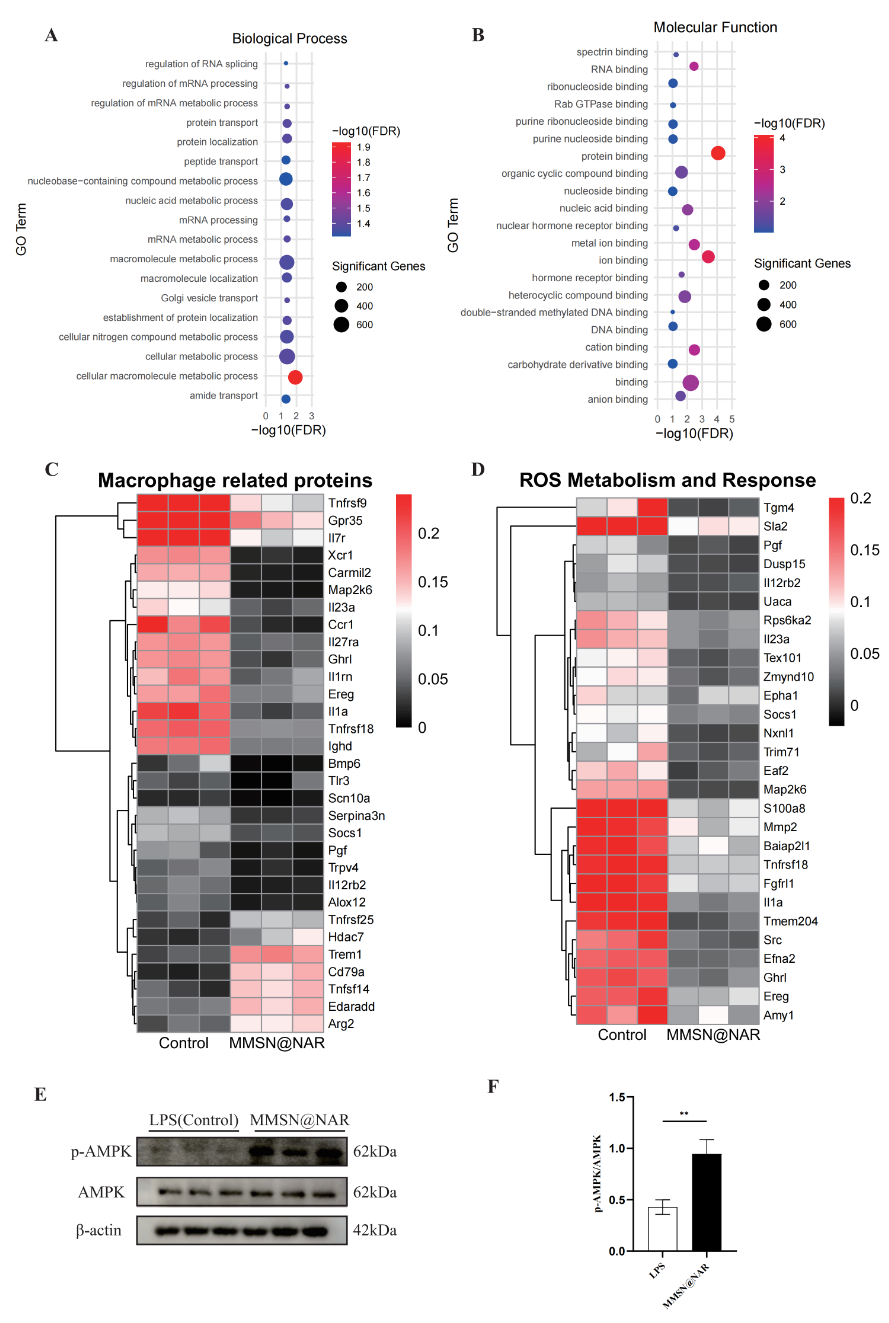


Supplementary Fig.2. Bioinformatics analysis of IL-1Ra@MMSN effects on macrophages. A) Clustering of enriched GO-biological process (BP) for DEG. The GO-BP terms with FDR＜0.05 are shown. B) Clustering of enriched GO-molecular function (MF) for DEG. The GO-MF terms with FDR＜0.10 are shown. C) Biological insight into the RAW264.7 cells treated with MMSN@NAR. Heatmaps reveal key biological processes in macrophage related proteins. D) Biological insight into the RAW264.7 cells treated with MMSN@NAR. Heatmaps reveal key biological processes in ROS metabolism and response. E) Western blot analysis of p-AMPK, AMPK protein levels in BMDMs. F) Semi-quantitative analysis of phosphorylated AMPK (Thr172) levels normalized to total AMPK protein expression (n=3).0.0021≦**p＜0.0332.


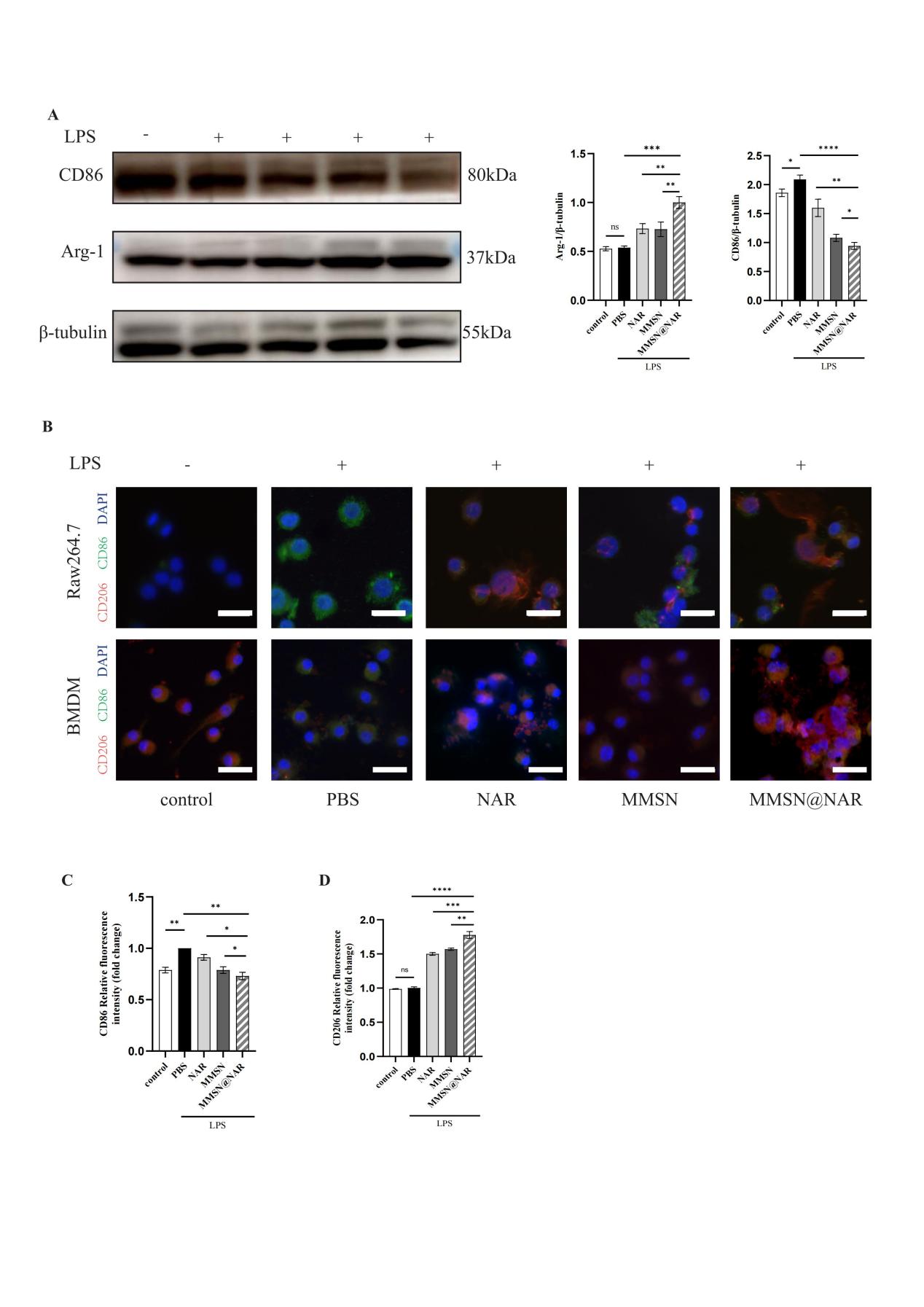


Supplementary Fig.3. MMSN@NAR-mediated macrophage polarization and ROS elimination in vitro. A)Western blot analysis of iNOS, Arg-1 protein levels in Raw264.7 macrophages. And Semiquantitative analysis of Arg-1 and CD86 expression levels in the indicated groups (n = 3). B) Immunofluorescence images of Raw264.7 and BMDM cells treated with LPS for 8 hours, followed by induction with PBS, naringenin, MMSN, or MMSN@NAR for an additional 8 hours. CD206 (red), CD86 (green), and nuclei stained with DAPI (blue). The scale bar represents 20 µm.C) Relative fluorescence intensity of CD86. D) Relative fluorescence intensity of CD206. 0.0332≦*p＜0.05, 0.0021≦**p＜0.0332, 0.0002≦***p＜0.0021, ****p＜0.0001.


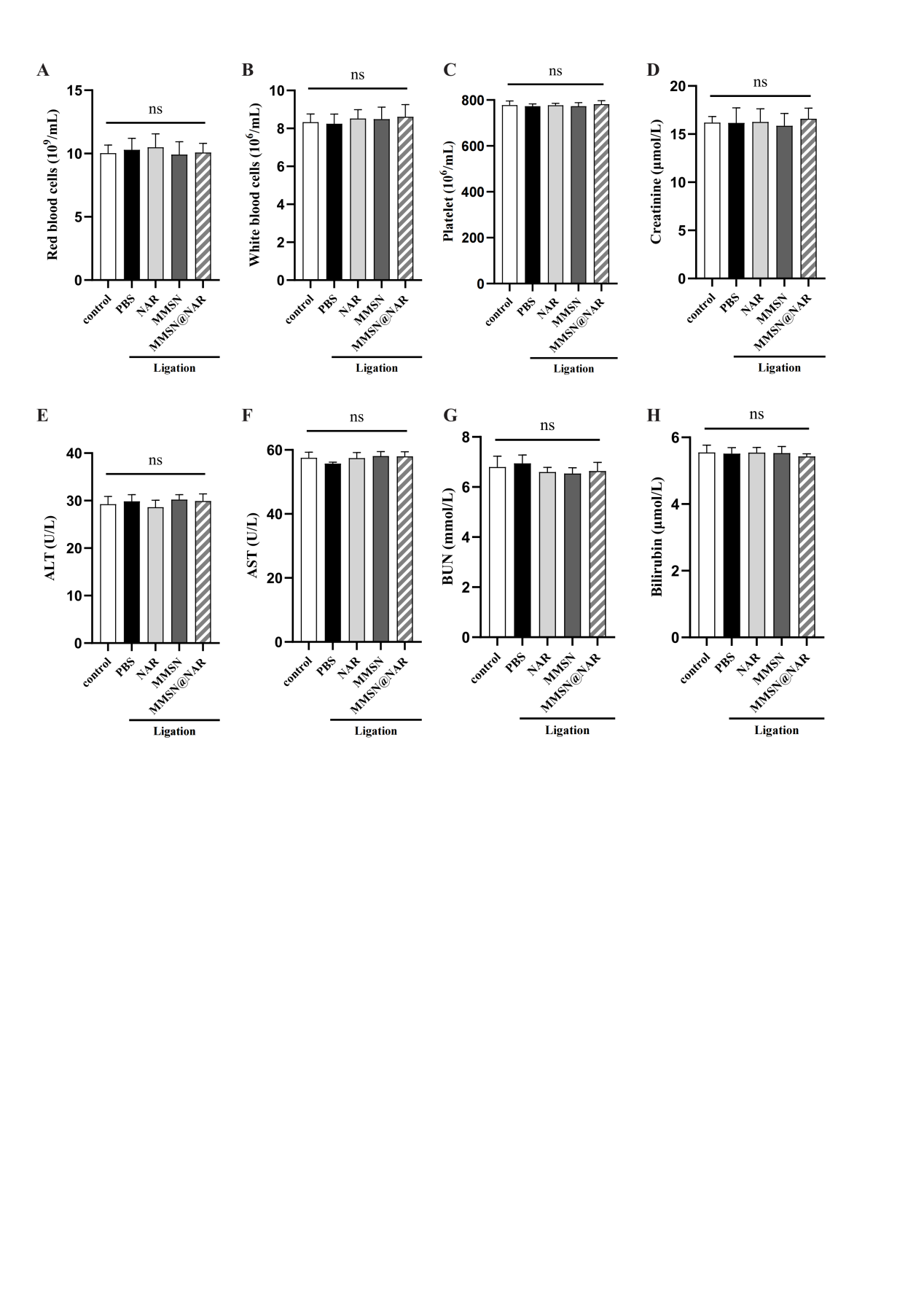


Supplementary Fig.4. Analysis of blood parameters, liver function, and kidney function in the specified experimental groups of mice. A) Red blood cell counts (n=6). B) White blood cell counts (n=6). C) Platelet counts (n=6). D) Creatinine levels (n=6). E) Lanine aminotransferase (ALT) levels (n=6). F) Aspartate aminotransferase (AST) levels (n=6). G) Blood urea nitrogen (BUN) levels (n=6). H) Bilirubin levels (n=6). NS indicates not significant.


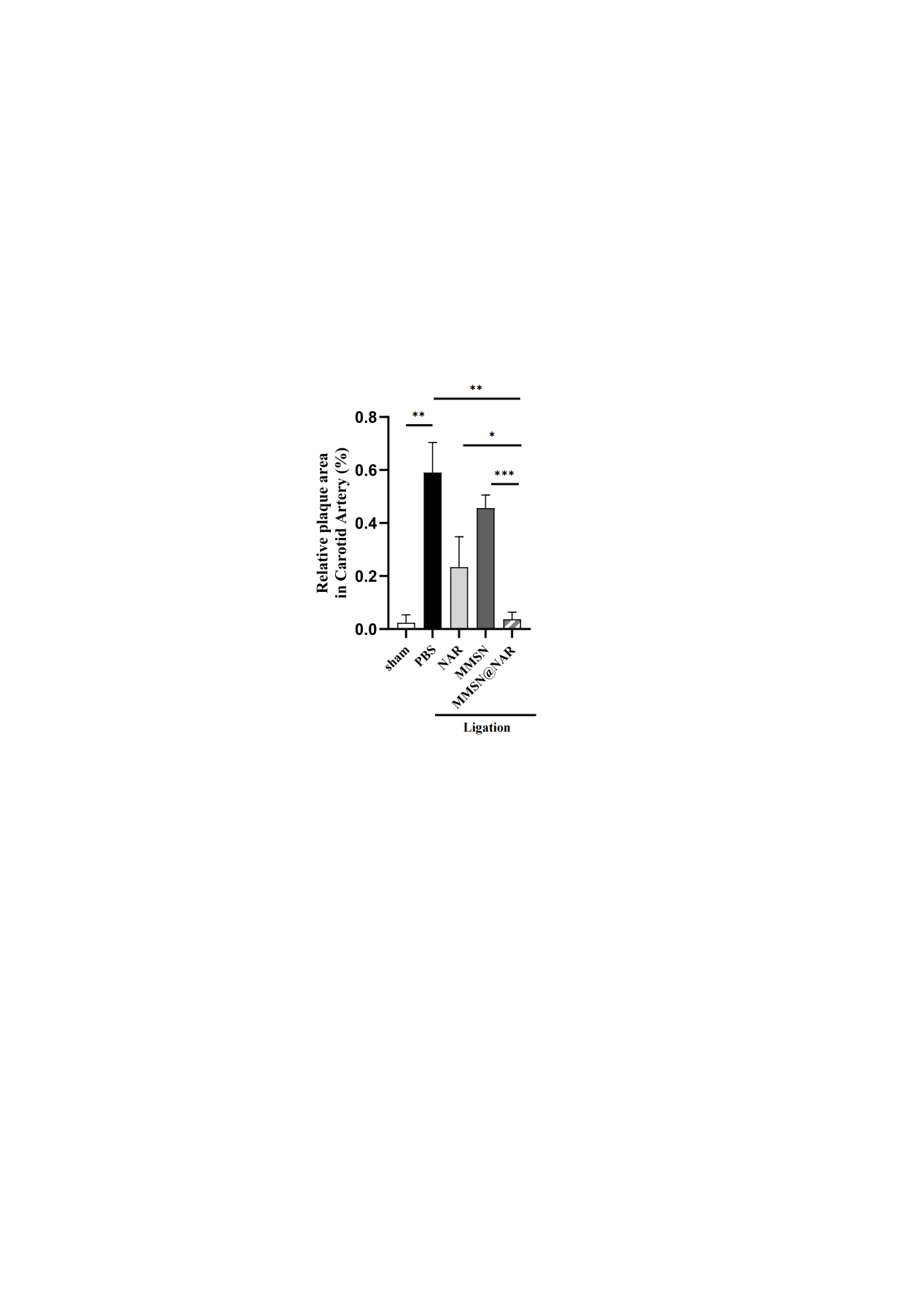


Supplementary Fig.5. The relative percentage area of plaque occupying the carotid artery lumen in specific groups (n=6). 0.0332≦*p＜0.05, 0.0021≦**p＜0.0332, 0.0002≦***p＜0.0021.

、


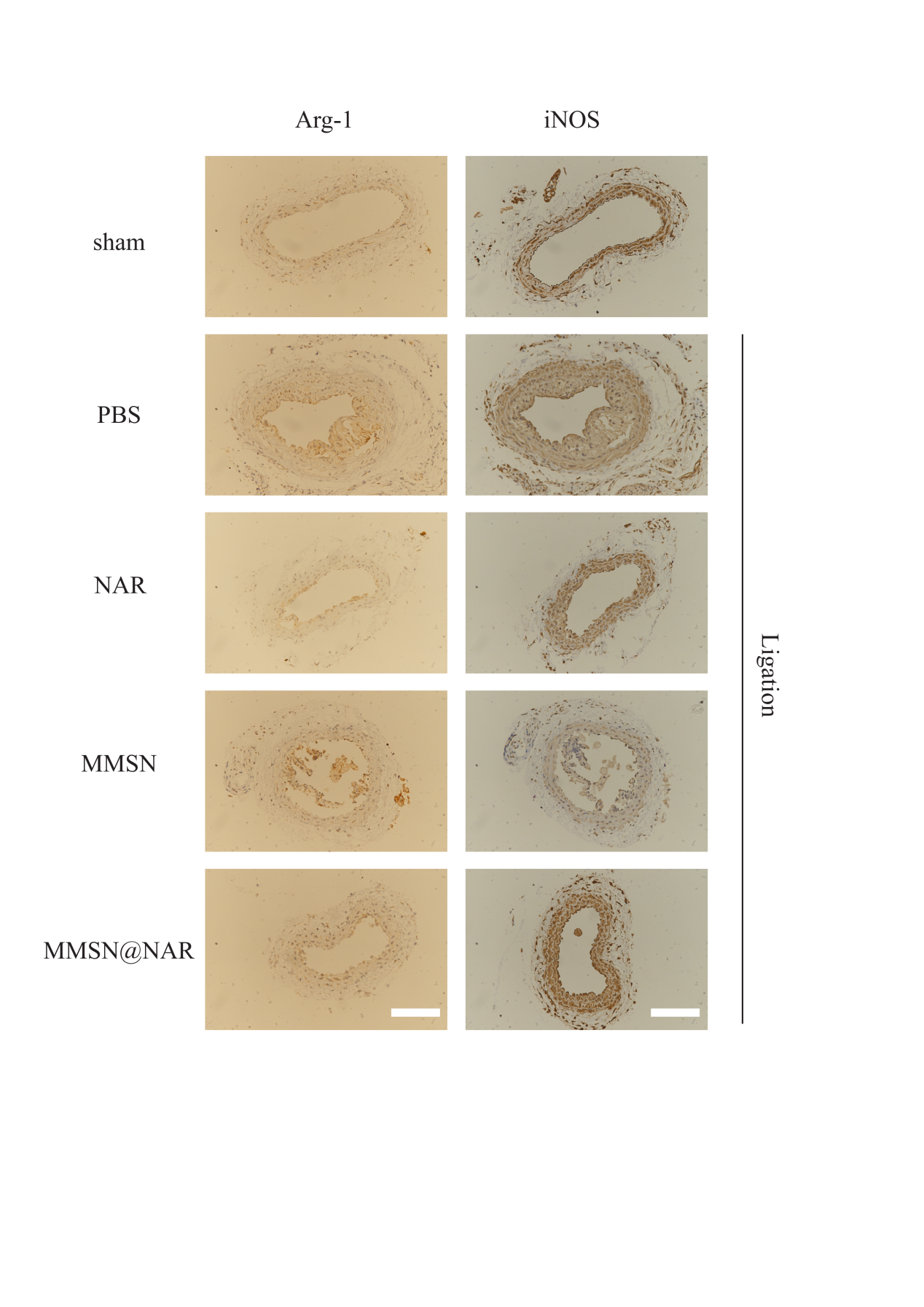


Supplementary Fig.6. Representative immunohistochemical images of Arg-1 and iNOS in plaque sections from the sham group (no ligation), and the PBS, naringenin, MMSN, and MMSN@NAR groups (all subjected to ligation surgery) (n=3). Scale bar: 200 µm.
